# Predicting polypharmacy in half a million adults in the Iranian population: comparison of machine learning algorithms

**DOI:** 10.1186/s12911-023-02177-5

**Published:** 2023-05-05

**Authors:** Maryam Seyedtabib, Naser Kamyari

**Affiliations:** 1 Department of Biostatistics and Epidemiology, School of Health, Ahvaz Jundishapur University of Medical Sciences, Ahvaz, Iran; 2 Department of Biostatistics and Epidemiology, School of Health, Abadan University of Medical Sciences, Abadan, Iran

**Keywords:** Polypharmacy, Machine learning, Artificial intelligence, Random Forest, Iranian

## Abstract

**Background:**

Polypharmacy (PP) is increasingly common in Iran, and contributes to the substantial burden of drug-related morbidity, increasing the potential for drug interactions and potentially inappropriate medications. Machine learning algorithms (ML) can be employed as an alternative solution for the prediction of PP. Therefore, our study aimed to compare several ML algorithms to predict the PP using the health insurance claims data and choose the best-performing algorithm as a predictive tool for decision-making.

**Methods:**

This population-based cross-sectional study was performed between April 2021 and March 2022. After feature selection, information about 550 thousand patients were obtained from National Center for Health Insurance Research (NCHIR). Afterwards, several ML algorithms were trained to predict PP. Finally, to assess the models’ performance, the metrics derived from the confusion matrix were calculated.

**Results:**

The study sample comprised 554 133 adults with a median (IQR) age of 51 years (40 – 62) that nested in 27 cities within the Khuzestan province of Iran. Most of the patients were female (62.5%), married (63.5%), and employed (83.2%) during the last year. The prevalence of PP in all populations was about 36.0%. After performing the feature selection, out of 23 features, the number of prescriptions, Insurance coverage for prescription drugs, and hypertension were found as the top three predictors. Experimental results showed that Random Forest (RF) performed better than other ML algorithms with recall, specificity, accuracy, precision and F1-score of 63.92%, 89.92%, 79.99%, 63.92% and 63.92% respectively.

**Conclusion:**

It was found that ML provides a reasonable level of accuracy in predicting polypharmacy. Therefore, the prediction models based on ML, especially the RF algorithm, performed better than other methods for predicting PP in Iranian people in terms of the performance criteria.

## Introduction

Polypharmacy (PP) refers to the ‘‘administration of many drugs simultaneously and/or the administration of more drugs than is clinically indicated, representing an unnecessary use of drug” [1]. The global definition is available for PP considering the actual number of drugs taken by one person, and in recent studies, intake of five or more medications is a commonly used definition of PP [2]. The prevalence of PP has been investigated in most studies among the elderly (≥ 65 years) population, and the data related to adults (≥ 18 years) have received less attention. The simultaneous use of multiple prescription drugs is increasingly common, with 27% of the population and 38% of adults in Iran using five or more medications at the same time [3]. Similarly high prevalence among adults is reported in other countries (e.g., 51.5% in Kingdom of Saudi Arabia [4], 36.8% in the United States [5], 22.4% in Poland [6], 30.7% in Scotland [7], 24.4% in Sweden [8], 39.1% in Germany [9], and 45.8% among Covid-19 patients [10]).

A great majority of studies on PP have focused on its potentially negative consequences, e.g., inappropriate prescribing, higher health care costs, non-compliance to medications, drug interactions, adverse drug reactions, decreased physical functioning, and quality of life [2, 5–7, 11, 12]. Some researchers have also investigated the prevalence of PP in the elderly or patients with chronic disease populations [13] and, the factors and conditions leading to PP have received in new studies [14–16]. To our knowledge, no study so far has analysed possible predictors for polypharmacy in patients consuming multiple drugs by new statistical classification methods.

Machine learning (ML) is becoming necessary for solving issues in many scopes, including healthcare [17]. Currently, we are seeing the introduction of various ML methods in different healthcare fields that can help professionals in the improvement of diagnosis [18–20]. An example is the use of a four-model ensemble strategy to categorise the probability of death of patients contaminated with COVID -19 [21]. Similarly, the clinical decision support system (CDSS) was developed to reduce prescribing errors by helping to prioritise the review of prescriptions [22, 23]. Similar support systems can be developed to help pharmaceutical companies select a suitable molecule with which to conduct research and which is likely to go through the approval process and reach the market [24]. Maternal health initiatives can use the CDSS to predict ectopic pregnancies [25]. Pharmaceutical companies are turning to machine learning to facilitate drug discovery and manufacturing. For its part, the FDA has proposed certain regulations that allow the use of AI and machine learning in medical devices. [26].

Despite the new studies in assessing PP [27], its modelling has still received less attention. Hence, we compared the performance of five ML methods in predicting PP in more than 5 thousand Iranian people to find the most favourable features and methods for our data.

In next section, we describe the required datasets and the details of ML algorithm. In results section, determination of the ML model are compared using the metrics derived from the confusion matrix. The conclusion and some possible further works are presented in Discussion Section.

## Materials and methods

### Data collection and preparation

A retrospective cohort study was conducted on health insurance claims data from April 2021 to March 2022, provided by National Center for Health Insurance Research (NCHIR) for elderly in Khuzestan province, Iran, which manages “Bimeh Salamat” for Iranians. As of March 2022, the insurance program was covering 554 133 beneficiaries from 27 cities in Khuzestan province.

The data include patients’ clinical and demographic characteristics, like age (≥ 18 years), sex (female, male), marital status (married, single), occupation (employed, unemployed), income (low, middle, high), residence area (rural, urban), ethnicity (Arab, Fars, Lor, Tork & Kord), and prescription’s variables per last 12 months include: number of prescriptions (NOP), number of drugs (NOD) per prescription, season of prescription (season), insurance coverage for prescription drugs (ICPD), total pharmaceutical spending (TPS $), number of visits to the general practitioner (NVGP), number of visits to a specialist (NVS). In addition, commonest non-communicable diseases (NCDs) in the subjects were selected by using International Classification of Diseases (ICD) codes, such as Diabetes mellitus (DM); Dyslipidemia (DLP); Asthma; Gastrointestinal reflux disease (GERD); Hypertension (HTN); Cardiovascular diseases (CVD) include heart failure, ischemic heart disease, arrhythmia, and stroke; Chronic kidney disease (CKD); Rheumatoid arthritis (RA) include rheumatoid arthritis and osteoarthritis; and Mental health conditions (MHC) include dementia anxiety and depression. It is worth noting that in the US, the number of prescriptions is usually the same as the number of drugs, so in the case of Iran, one prescription may contain several drugs.

All variables (24 variables) in patients’ records were extracted and regarded. Normalization of the continuous variables was done. The outcome was binary PP that was calculated from NOD. Using the SMOTE method, handling the imbalanced dataset problem was done. The research protocol was approved by the Ethics Committee of the Abadan University of Medical Science (No. IR.ABADANUMS. REC.1401.101).

Certain classes were clustered to reduce the number of classes of these variables. Records, which had over 70% of missing data, were not included in the analysis. The imputation technique was used for the remaining missing values, assuming that the missing data had a random distribution, [28]. Little’s MCAR test evaluated MCAR with the null hypothesis that the data are missing completely at random (MCAR) [29].

### Predictor variables

The analysis was done on data in three classes of predictor variables obtained from the health insurance claims data. Twenty-three variables were classified as socio-demographic characteristics (seven), prescriptions (six), and comorbidities (ten).

### Outcome variable

There is no unique consensus on the PP definition. As reported earlier, PP is defined as the concomitant prescription of five or more medications per prescription [3, 30]. The feature demonstrates the class variable, which is binary. For each patient, if the average number of prescribed drugs (NOD) per prescription/year is less than five, then PP is 0; otherwise, it will be 1. Out of the 554 133 patients, 199 485 instances were labeled as 1 (Table 1).

**Table 1:** Definitions of evaluation metrics

Performance measures	Definitions
Accuracy	(TP + TN)/(TP + FP + FN + TN)
Precision	TP/(TP + FP)
Recall/ Sensitivity	TP/(TP + FN)
Specificity	TN/(TN + FP)
F1-score	(2 × TP)/(2 × TP + FP + FN)

True positive (TP), true negative (TN), false positive (FP), false negative (FN)

### Data balancing

The imbalanced data problem is an important barrier to ML algorithms, which can be seen due to no equal categorization of the classes. In a considered dataset, the data amount in outcome classes is markedly imbalanced containing more samples associated with the non-polypharmacy class (64.0%), whereas the PP class is much smaller (36.0%). Therefore, the trained models usually provide biassed results for the predominant class and the ML models assign new observations to the majority class. We applied the edited nearest neighbor (ENN) along with synthetic minority over-sampling technique (SMOTE) to deal with the class imbalance in the imbalanced-learn toolbox to make the dataset balanced (SMOTEENN 0.9.1).

### Feature selection

The feature selection improves the performance of a predictive model and reduces the modeling computational cost by selecting the most important variables; therefore, it reduces the computational complexity of the model. Another goal was to gain insight into the underlying processes, which generated the data [31, 32]. Therefore, prior to model prediction, feature selection should be done. Through the calculation of different ML algorithms and the removal of irrelevant factors, errors were reduced in clinical decisions and accuracy improved [32]. To indicate the best predictors, the effectiveness of different feature selection methods was compared. Therefore, in the training set, five methods including eXtreme Gradient Boosting (XGBoost), Decision Tree (DT), Support Vector Machine (SVM), Random Forest (RF), and Artificial Neural Networks (ANN’s) were applied to train through the selection of the relevant features for to predict PP. To prevent overfitting, the ten-fold cross-validation was applied in the training process.

### Model development

We trained five ML algorithms, namely DT, RF, XGBoost, SVM, and ANN in the “*Rattle*” (R Analytical Tool to Learn Easily) package application. Rattle is used for data mining written in R and provides a Graphical Data Interface [33]. To implement these models, we experimentally matched the hyperparameters to the training split of the dataset based on cross-validation (CV). A standard ML technique called k-fold cross-validation (tenfold in our study) was used to train and test ML models. Each method is described below.

### Decision trees

DT induction is a classic ML technique that is deployed in data mining [34]. It is very effective as it uses a simple algorithm and a simple tree structure for representing the model. DT can be regarded as a series of IF–THEN rules as well as as conditional probability distributions defined in class and feature spaces [34, 35]. When the samples are in one class, the node can become the leaf and is marked by the class. Otherwise, the algorithm selects the discriminatory attribute as the DT current node [36]. Based on the current decision node attribute value, the training samples can be categorized into many subsets and each forms a branch. For every obtained branch or subset, the previous stages should be repeated, recursively producing a DT on each partitioned sample [37–39]. Such induction structure is simple for interpretation, easy to implement due to less complicated calculations, and does not need data normalization [40, 41]. The *rpart* package is employed to form the DT.

### Random forest

The RF was proposed by Breiman and has many individual DTs that work together as a group [42]. It boosts accuracy using a group of decision models instead of a single learning model. The important difference between this technique and traditional DT algorithms is splitting nodes of the root nodes that are generated randomly [43]. The trees are protective each other against their defects leading to their strong effect. Some trees may estimate wrong classification, but several others are correct, leading to progression in an appropriate direction. Therefore, the predictions and errors caused by particular trees should be correlated with each other; thus, the RF can perform well [44]. Moreover, RF has several advantageous, like being used for both regression and classification duties and processing missing variables. In addition, overfitting occurs less when more DT are added to the forest [45–47].

### eXtreme gradient boosting

Chen et al. proposed XGBoost method in 2016 [48], which is an ensemble approach based on DT method. XGBoost is an open-source library and is presented as a scalable tree boosting system. It is built on DT models. After introducing the trees to the ensemble one they are fitted to make prediction mistakes correct due to previous models and then the prediction is made [37, 38]. The gradient boosting framework is used and models are added sequentially. Hence, it is capable of minimizing errors, maximizing models’ performance, and reducing tree construction length [49]. XGBoost is deployed on many challenges, and can produce state-of-the-art outcomes on many difficult problems [50]. It is extremely and computationally (fast to execute) effective. The *xgboost* package is used to build the boosted model.

### Support vector machine

The SVM method was first introduced by *Stephan R. Sain* and *V.N. Vapnik* based on statistical learning theory [51]. SVM was designed for twofold classification. However, it is effectively expanded for multi-class situations. SVM finds a line/ hyper-plane in a multidimensional space capable of splitting the feature space into specific groups [52–54]. The “*kernel*” is the main SVM algorithm. Data that cannot be linearly divided into lower dimensions are transferred by the kernel to a higher dimension. This SVM capacity causes its good performance than other techniques [55–57]. SVR is an extension of SVM, which is used for regarding the risk of structural, reducing the generalization error, and increasing hyper-plane margin to decrease the tolerated error [58, 59]. *Rattle* deploys *ksvm* from the *kernlab* package.

### Artificial neural networks

Neural Networks introduced by Warren McCulloch and Walter Pitts in 1943 as an old method for modeling can imitate a human's neural network and were designed considering the central nervous system [60]. A neural network as a non-parametric regression approach has a series of highly interconnected nodes to model complex functions [61, 62]. ANN like the biological neural network is generated by nodes, neurons, or processing features that are connected to make a network. The ANN accumulates data from all surrounding neurons and offers an output associated with its activation functions and weight. Adaptive weights can indicate the strong points of the connection between neurons. To perform the learning process, they must be adjusted so that the network output is nearly similar to the favorable output. Mathematically, this can be well described in a fairly simple, if not straightforward, way. *Rattle* employs the functionality offered by the *nnet* package.

### Cross‑validation

The k-fold cross-validation (k-fold CV) works based on repeated holdout. It has become the initiative standard to estimate the performance of the model. Instead of repeated random sampling, k-folds CV can randomly divide the data into folds [63]. To assess the algorithm performance, ten-fold CV was applied to evaluate predictive models and obtain reliable findings. Using stratified random sampling, the main training dataset was divided into ten folds (each comprising 10 percent of the total data). For each of the 30 percent of data, a ML model is formed on the remaining 70% of data. The fold's 30% sample evaluates the model. Following training and evaluating for 100 times (with 100 various training/testing combinations), the mean performance is reported. The whole samples in the dataset can be trained and evaluated, leading to no higher variance [64, 65]. Datasets for cross-validation are formed by the *createFolds* function in the *caret* package.

### Model evaluation

Evaluation of the model performance is a virtual stage of producing a useful ML model, which is done using some performance indices, mostly obtained from the confusion matrix. In this study, recall, accuracy, specificity, precision, and F1-score metrics were used to compare the performance of methods on validation and training sets in each cross-validation iteration (Table 1). The interpretation for all measures were *poor* < 50%, *OK*: 50–80%, *good*: 80–90%, and > 90% *very good*. Such criteria are mostly reported in the model evaluation using ML techniques [66]. The *caret* (Classification and Regression Training) package by Max Kuhn has functions to compute several performance measures. It offers many tools for training, preparing, visualizing and evaluating ML models and data [67].

**Fig. 1:**
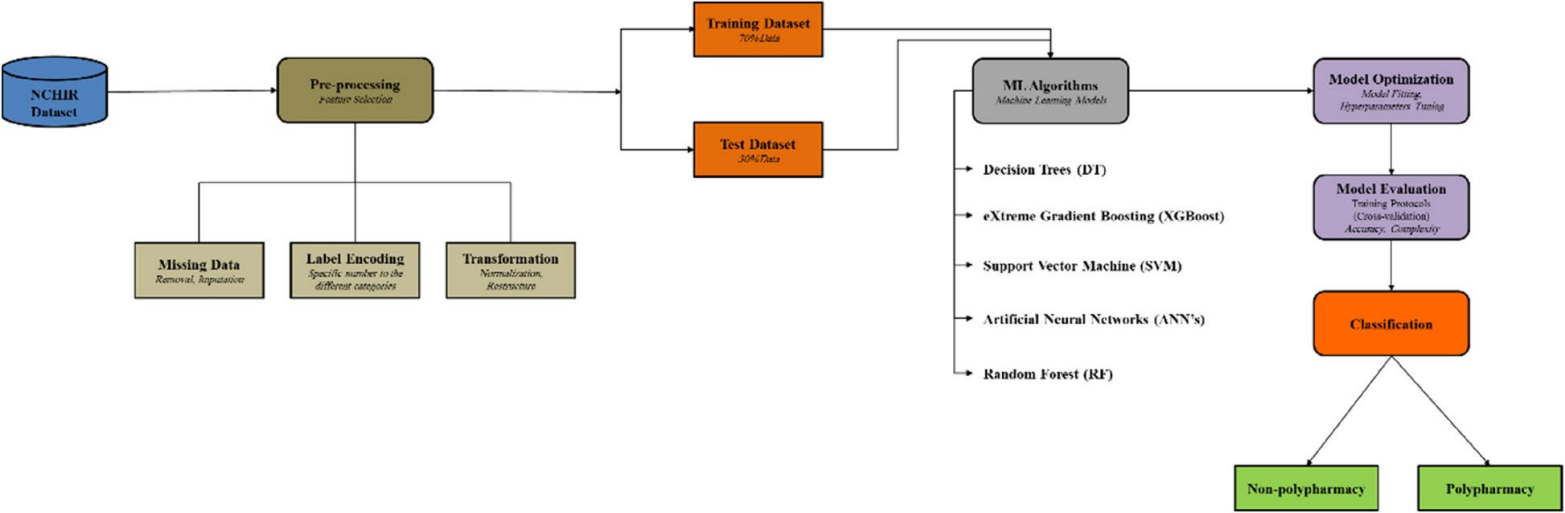
Workflow Diagram of Proposed Method of Polypharmacy’s Classification

Although different types of ML will have distinct approaches to training the model, there are basic steps that most models utilize [68]. Figure 1 gives an overview of the process of the steps taken to create the Machine Learning models in the prediction of PP.

## Results

### Patient characteristics

The study sample comprised 554 133 adults (≥ 18 years) males and females that nested in 27 cities within Khuzestan province of Iran and their characteristics are provided in Table 2. The median (IQR) age of the patients was 51 years (40 – 62). Among the patients, 62.5% (n = 346 569) were female, 63.5% (n = 351 875) were married and 83.2% (n = 461 039) were employed during the last year (2021 – 2022). In total, 23.3% (n = 129 312) of people were with high-income level, 21.5% (n = 119 294) of people were living in rural areas and the majority were Arab ethnicity (33.3%).

**Table 2:** Characteristics of samples

**Variable**	**Total** **(*n* = 554 133)**	**Polypharmacy**	
		**No** **(*n* = 354 648)**	**Yes** **(*n* = 199 485)**
***Socio-demographic***			
Age (year); Median (IQR)	51 (40 – 62) yr	51 (39 – 61) yr	52 (41 – 63) yr
Gender			
Female	346 569 (62.5%)	221 554 (62.5%)	125 015 (62.7%)
Male	207 564 (37.5%)	133 094 (37.5%)	74 470 (37.3%)
Marital status			
Single	202 258 (36.5%)	131 574 (37.1%)	70 684 (35.4%)
Married	351 875 (63.5%)	226 265 (63.8%)	125 610 (63.0%)
Employment status			
Employed	461 039 (83.2%)	294 358 (83.0%)	166 681 (83.6%)
Unemployed	93 094 (16.8%)	61 354 (17.1%)	31 740 (15.9%)
Income			
Low	231 384 (41.8%)	148 598 (41.9%)	82 786 (41.5%)
Middle	193 578 (34.9%)	123 559 (34.8%)	70 019 (35.1%)
High	129 312 (23.3%)	82 633 (23.3%)	46 679 (23.4%)
Residence area			
Urban	434 839 (78.5%)	278 044 (78.4%)	156 795 (78.6%)
Rural	119 294 (21.5%)	76 604 (21.6%)	42 690 (21.4%)
Ethnicity			
Arab	184 614 (33.3%)	117 388 (33.1%)	67 226 (33.7%)
Fars	179 450 (32.4%)	115 615 (32.6%)	63 835 (32.0%)
Lor	167 548 (30.2%)	107 104 (30.2%)	60 444 (30.3%)
Tork & Kord	22 521 (4.1%)	14 541 (4.1%)	7 980 (4.0%)
***Prescriptions***			
Season			
Spring	132 991 (24.0%)	85 115 (24.0%)	47 876 (24.0%)
Summer	131 130 (23.7%)	84 052 (23.7%)	47 078 (23.6%)
Autumn	146 491 (26.4%)	93 627 (26.4%)	52 864 (26.5%)
Winter	143 521 (25.9%)	91 854 (25.9%)	51 667 (25.9%)
TPS $; Median (IQR), per persc/y	4.4 (2.1 – 10.3) $	2.9 (1.5 – 6.7) $	7.7 (4.4 – 14.8) $
NOP; Median (IQR), per year	2.7 (1.4 – 4.3)	2.6 (1.4 – 4.3)	2.7 (1.5 – 4.4)
NOD; Median (IQR), per persc/y	4.7 (2.1 – 6.4)	3.8 (2.5 – 5.6)	4.2 (3.7 – 7.1)
ICPD $; Median (IQR), per persc/y	1.3 (0.9 – 1.8)$	1.3 (0.9 – 1.8)$	1.4 (0.9 – 1.9)$
NVGP; Median (IQR), per year	2.2 (1.4 – 4.6)	2.1 (2.0 – 3.9)	2.2 (2.0 – 4.1)
NVS; Median (IQR), per year	2.3 (1.5 – 4.7)	2.2 (1.9 – 3.7)	2.3 (2.1 – 4.1)
***Comorbidity***			
Any comorbidity	169 942 (30.7%)	107 104 (30.2%)	62 838 (31.5%)
DM	47 943 (8.7%)	29 790 (8.4%)	18 153 (9.1%)
Asthma	46 592 (8.4%)	29 436 (8.3%)	17 156 (8.6%)
DLP	363 135 (65.5%)	230 876 (65.1%)	132 259 (66.3%)
HTN	209 751 (37.9%)	130 156 (36.7%)	79 595 (39.9%)
GERD	239 518 (43.2%)	152 144 (42.9%)	87 374 (43.7%)
CVD	30 699 (5.5%)	17 732 (5.0%)	12 967 (6.9%)
CKD	47 079 (8.5%)	28 726 (8.1%)	18 353 (9.2%)
RA	4 581 (0.8%)	2 766 (0.8%)	1 815 (0.9%)
MHC	119 892 (21.6%)	76 604 (21.6%)	43 288 (21.7%)

*TPS* total pharmaceutical spending, *NOP* number of prescriptions, *NOD* number of drugs, *ICPD* insurance coverage for prescription drugs, *NVGP* number of visits to the general practitioner, *NVS* number of visits to a specialist, *DM* diabetes mellitus, *DLP* dyslipidemia, *HTN* hypertension, *GERD* gastrointestinal reflux disease, *CVD* cardiovascular diseases, *CKD* chronic kidney disease, *RA* rheumatoid arthritis, *MHC* mental health conditions

From the previous 12 months, the most number of visits was in the autumn (26.4%) and the least number of people's visits were in the summer (23.7%). The average price of drugs for Khuzestan residents was 4.4 (IQR = 2.1 – 10.3) dollars per prescription and the average number of drugs was 4.1 (IQR = 2.1 – 6.4) per prescription. ICPD were about 30% (1.3/4.4 = 0.299) of total cost. The median number of persecutions for each person was 2.7 (IQR = 1.4 – 4.3) per year. On average, Khuzestan residents had two general practitioners visit and two specialist visits per year.

More than 30% of people have been suffering from any of the underlying medical conditions. The comorbidities variables included the following: diabetes (8.7%), asthma (8.4%), dyslipidemia (65.5%), HTN (37.9%), gastrointestinal reflux disease (43.2%), cardiovascular diseases (5.5%), chronic kidney disease (8.5%), rheumatoid arthritis (0.8%), and mental health conditions (21.6%).

### Developing and evaluating models

After selecting the best subset of features, various ML algorithms were used to build the predictive model. Five ML algorithms, such as DT, XGBoost, SVM, ANN’s and RF, were trained to develop PP prediction models and their performance was assessed through sensitivity (recall), precision, accuracy, specificity, and F1-score of the performance metrics.

**Fig. 2:**
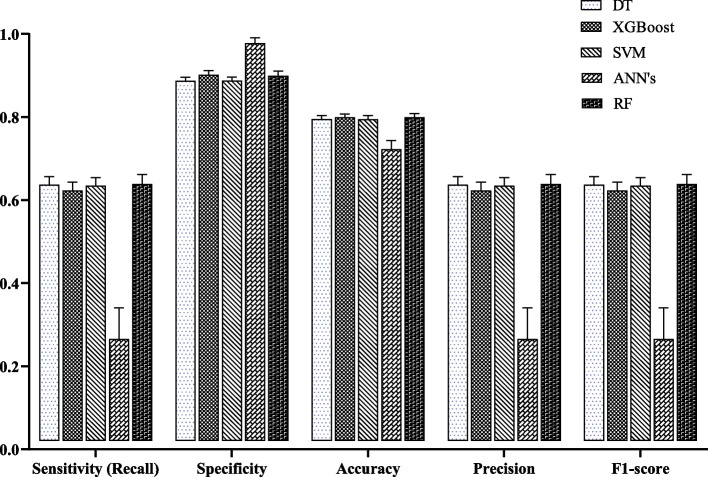
The average performance metrics of the selected ML algorithms for test set. DT: Decision Tree; XGBoost: eXtreme Gradient Boosting; SVM: Support Vector Machine; ANN’s: Artificial Neural Networks; RF: Random Forest

Table 3 shows their discriminative capacity for predicting PP in training and test sets. The RF method performance, regarding recall, accuracy, precision, and F1-score, was higher for training and test sets. According to the test set, the specificity and accuracy of XGBoost were similar to that of RF (spe = 90.2% & acc = 79.94%). ANN’s had the highest specificity among the ML methods (98.82%). ANN’s and RF had the lowest and highest values in sensitivity, accuracy, precision and F1-score, respectively. The average accuracy of the ML methods was from 72.23% to 79.99% for the test sets and ANN’s and RF showed the lowest and highest values, respectively. Also, the average specificity of all ML methods was more than 88%. Figure 2 displays the average performance indices of the considered ML algorithms for test set.

**Table 3:** Performance criteria of ML methods for polypharmacy prediction

Model	Set	Sensitivity (Recall)%	Specificity%	Accuracy%	Precision%	F1-score%
DT	Train	63.76 (0.82)	88.67 (0.37)	79.46 (0.38)	63.76 (0.82)	63.76 (0.82)
	Test	63.69 (1.92)	88.70 (0.86)	79.50 (0.89)	63.69 (1.92)	63.69 (1.92)
XGBoost	Train	66.00 (0.97)	92.26 (0.45)	82.55 (0.38)	66.00 (0.97)	66.00 (0.97)
	Test	62.31 (1.97)	90.20 (1.00)	79.94 (0.80)	62.31 (1.97)	62.31 (1.97)
SVM	Train	63.80 (0.84)	88.97 (0.39)	79.66 (0.38)	63.80 (0.84)	63.80 (0.84)
	Test	63.46 (1.95)	88.81 (0.86)	79.49 (0.90)	63.46 (1.95)	63.46 (1.95)
ANN’s	Train	27.63 (6.70)	99.01 (1.06)	72.61 (1.82)	27.63 (6.70)	27.63 (6.70)
	Test	26.52 (7.53)	98.82 (1.27)	72.23 (2.18)	26.52 (7.53)	26.52 (7.53)
RF	Train	69.85 (1.37)	92.87 (0.42)	84.34 (0.46)	69.85 (1.37)	69.85 (1.37)
	Test	63.92 (2.27)	89.92 (1.14)	79.99 (0.88)	63.92 (2.27)	63.92 (2.27)

Averages are expressed as the Mean (SD)

*DT* Decision Tree, *XGBoost* eXtreme Gradient Boosting, *SVM* Support Vector Machine, *ANN’s* Artificial Neural Networks, *RF* Random Forest

**Fig. 3:**
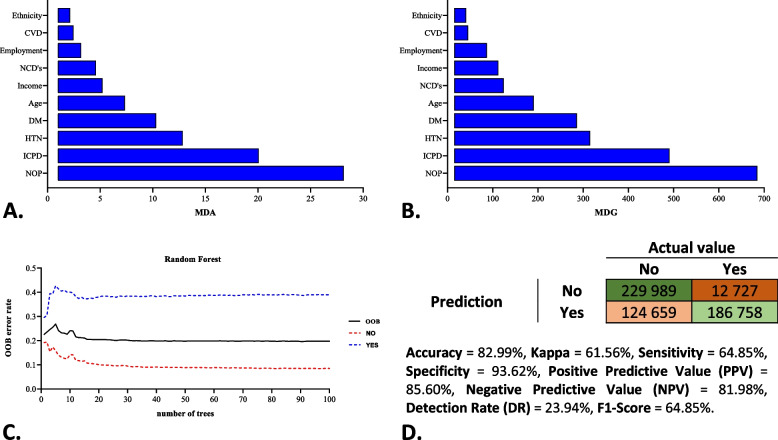
Top 10 variable importance (VIMP) values for predicting polypharmacy in Khuzestan residence patients using: **A** mean decrease in classification accuracy (MDA) or **B** mean decrease in classification Gini impurity (MDG); **C** The error rate of the RF model (OOB: out of bag, 0: Non-polypharmacy, and 1: Polypharmacy); **D** Confusion matrix performance metrics. The results was an average of 100 runs of RF. NOP: Number of prescriptions; ICPD: Insurance coverage for prescription drugs; NCD’s: Non-communicable diseases; DM: Diabetes mellitus; HTN: Hypertension; CVD: Cardiovascular diseases

Figure 3 indicates the top ten VIMPs derived from RF using test and training sets (all dataset). Ranking of the variables is done using the average of 100 runs on the average reduction in classification accuracy (MDA) or the average reduction in classification Gini impurity (MDG). Ranking of all 24 variables was done using their MDA and MDG to classify the subjects into PP or non-PP categories. The ten most crucial variables were recognized according to MDG, which is highly stable during classification permutation. NOP, ICPD, and HTN were the three most crucial variables to predict PP in patients. Among socio-demographic features age, income and employment status were most influential variables (Fig. 3A, B). The optimal classification was obtained through this set of ten variables, with an accuracy of 82.81% and out-of-bag (OOB) error rate of 19.84% (Fig. 3C, D).

## Discussion

PP as a complicated issue can differ in implications and inappropriateness for medically complex patients than those who are more beneficial. The predicts of PP include features associated with the patient (sociodemographic factors, like age, gender, income, place of residence, and ethnicity), the healthcare system or to the physician (prescribed drug information such as costs, number of prescription), as well as the disease (certain diseases, like hypertension or diabetes mellitus, multiple comorbidity status). How to accurately diagnose and predict PP using ML algorithms is valuable studying. Based on the mentioned experiments, except ANN’s, we found, the good performance of using DT, XGBoost, SVM, and RF, and the results of using important features have worth findings.

PP is common in adults, especially females, elderly, and cases with comorbidities. Considering the adverse outcomes of PP, the prevalence of PP and its related features PP should be understood. Patients should be regularly assessed by clinicians for the presence of PP and institute measures to decrease inappropriate PP if possible.

In our study, among the socio-demographic features, age, income and employment status were the most influential variables. Taherifard et.al. studied the population-based prevalence of polypharmacy and patterns of medication use in southwestern Iran and found that socioeconomic status was not associated with polypharmacy but was significantly associated with patterns of medication use for digestive, metabolic and nervous system diseases [16]. Doheny et al. in a population-based study aimed at examining sociodemographic differences in polypharmacy among the elderly, show that there were greater sociodemographic differences among independents, with those with less education, older age and women being more likely to have polypharmacy [69]. In our study, among the prescription features, number of prescriptions and prescription drug insurance coverage were found to be the two most important predictors. Akande et al. have shown in a cross-sectional study that taking too many prescription drugs, intentionally skipping pills because there are too many, and regularly taking prescriptions from more than one doctor are the most important factors associated with polypharmacy [70]. In many studies, chronic disease was associated with reduced odds of polypharmacy [69, 71, 72]. Mizokami et al. conclude that physicians should carefully consider the type of chronic disease when assessing the risk of polypharmacy. Older patients with multiple diseases may experience further polypharmacy [72]. In our study, NCDs, particularly HTN, DM, and CVD, were significantly associated with the odds of polypharmacy. A large randomised controlled multicentre trial was conducted by Almodovar et al. to analyse the characteristics of an elderly multimorbid population with polypharmacy. The results show that frailty, multimorbidity, obesity and reduced physical as well as mental health status are risk factors for excessive polypharmacy [71]. Finally, in this research as in Almodovar's study, gender and marital status are not associated with excessive polypharmacy [71].

The results of the comparison of machine learning algorithms showed that, regarding performance criteria, RF was more favorable compared to other ML methods to predict PP. Other ML approaches, except ANN’s, showed the same performance and OK discrimination (accuracy: 79.49% – 79.94%).

The ML can be used for analysis and inference in a large set of retrospective datasets to extract specific relationships or determine strange patterns with minimal human intervention or without programming effort [73]. Similarly, the techniques of ML can be used in medical practise to improve prognostic modelling and uncover new factors associated with a particular target outcome to predict future or obscure trends [74]. In medical imaging studies, for example, ML and deep learning help with COVID -19 diagnosis and provide non-invasive detection measures to prevent medical staff from becoming infected with pathogens [75]. In virological studies, ML is used to study the genetics associated with the SARS-CoV-2 protein and predict new combinations that can be used to produce drugs and vaccines [75]. This model can therefore also be used to predict PP.

Our main limitation was no features associated with physical activities, body mass index, health habits, nutrition patterns, and certain clinical data influencing the medication use and PP, and their related outcomes. However, we indicated that ML methods have good performance in predicting PP in Iranian population. Lengthening the running time of the programs due to the size of the sample (big data) was another limitation of this research.

## Conclusions

In this paper, we propose five ML models that predicts polypharmacy in an adult Iranian people. The models have trained on data of all individuals’ information in NCHIR of Khuzestan province by using data for the last 12 months. Results show that our model can be implemented globally for effective screening and prioritization of assessing polypharmacy in the general population. In conclusion, according to the all above experiments, we found that the RF performance provided better results compared to other ML methods for predicting PP in Iranian people. In addition, clinicians should know the common occurrence of PP and try to reduce improper prescribing or inappropriate PP if possible. In future studies, the proposed method can be used to predict polypharmacy in the elderly. Furthermore, the performance of our model will improve as we test more classification techniques on small and qualitative datasets.

## Data Availability

The datasets generated and/or analysed during the current study are not publicly available but are available from the corresponding author on reasonable request.

## Abbreviations

PP: Polypharmacy
ML: Machine learning
NCHIR: National center for health insurance research
AI: Artificial intelligence
RF: Random forest
NOP: Number of prescriptions
NOD: Number of drugs per prescription
ICPD: Insurance coverage for prescription drugs
TPS: Total pharmaceutical spending
NVGS: Number of visits to the general practitioner
NVS: Number of visits to a specialist
NCDs: Non-communicable diseases
DM: Diabetes mellitus
DLP: Dyslipidemia
HTN: Hypertension
GERD: Gastrointestinal reflux disease
CVD: Cardiovascular diseases
CKD: Chronic kidney disease
RA: Rheumatoid arthritis
MHC: Mental health conditions
MDA: Mean decrease in accuracy MDG: Mean decrease in Gini impurity
MCAR: Missing completely at random
SMOTE: Synthetic minority over-sampling technique
ENN: Edited nearest neighbor
DT: Decision Tree
XGBoost: EXtreme Gradient Boosting
SVM: Support vector machine
ANN’s: Artificial Neural Networks
CV: Cross‑validation
TP: True positive
TN: True negative
FP: False positive
FN: False negative
OOB: Out-of-bag
