# Correction to: Predicting polypharmacy in half a million adults in the Iranian population: comparison of machine learning algorithms

**DOI:** 10.1186/s12911-023-02203-6

**Published:** 2023-06-22

**Authors:** Maryam Seyedtabib, Naser Kamyari

**Affiliations:** Department of Biostatistics and Epidemiology, School of Health, Ahvaz Jundishapur University of Medical Sciences, Ahvaz, Iran

**BMC Medical Informatics and Decision Making (2023) 23:84**

10.1186/s12911-023-02177-5

The original publication of this article contained an incorrect ethics code. The incorrect and correct information is listed in this correction article. The original article has been updated.

Incorrect: IR.ABADANUMS. REC.1401.048

Correct: IR.ABADANUMS. REC.1401.101

